# The Association Between Glycolyzed Hemoglobin A1c and Hearing Loss in Diabetic Patients

**DOI:** 10.7759/cureus.10254

**Published:** 2020-09-05

**Authors:** Elif Elibol, Hacer Baran

**Affiliations:** 1 Otolaryngology, Ankara Yıldırım Beyazıt University, Yenimahalle Government Hospital, Ankara, TUR; 2 Otolaryngology, Istanbul Lutfi Kırdar City Hospital, Istanbul, TUR

**Keywords:** diabetes, hearing loss, pure tone audiometry, glycated hemoglobin (hba1c)

## Abstract

Purpose

Our study aimed to determine the correlation between glycated hemoglobin (HbA1c) levels and the audiometric parameters in diabetic patients.

Methods

We included 724 patients (376 male, 348 female) in our outpatient clinic and created four groups by reference to the HbA1c values. The first group was 192 patients with an HbA1c value < 4.5, 176 patients with an HbA1c value between 4.5 - 5 as Group 2, 177 patients with an HbA1c value between 5 - 6 as Group 3, and 179 patients that had an HbA1c value greater than 6 as Group 4. The correlations between HbA1c values and median pure tone thresholds at 250, 500, 1,000, 2,000, 4,000, and 8,000 Hz, the speech recognition thresholds, and the speech discrimination scores were compared.

Results

The median speech recognition thresholds, speech discrimination scores, and the median pure-tone thresholds at 250, 500, 1,000, 2,000, 4,000, and 8,000 MHz in the left ear significantly differed in the fourth group (p < 0.001). Pure-tone thresholds at 500 Hz, 4,000 Hz, speech discrimination scores, and recognition thresholds in the right ear had a significant positive correlation in the fourth group (p < 0.001).

Conclusion

According to HbA1c levels, the severity of diabetes can affect high to all frequencies of hearing functions negatively. The follow-up of patients with higher levels of HbA1c should undergo audiological tests more regularly.

## Introduction

Hearing is a vital part of daily life, and in case of loss, quality of life is significantly affected due to impaired communication and functional ability [1-2]. According to the data of the World Health Organization (WHO), approximately 360 million people, which constitutes 5% of the world population, experience a hearing loss problem, and this rate increases due to the higher elderly population [3]. Hearing loss in the adult population can develop due to age, genetic factors, neurological diseases, causes of vascular origin, metabolic disorders, ototoxic drugs, noise, and diabetes. Since age-related hearing loss cannot be reversed, it is important to identify preventable causes [4-6].

Diabetes is a chronic disease, and in the severe stage, it causes many organ disorders or deficiencies in patients. Cardiovascular and peripheral vascular diseases, neuropathy, nephropathy, retinopathy, and cerebrovascular disorders are among the long-term complications of diabetes [7]. Hemoglobin A1c (HbA1c) is formed by the non-enzymatic and slow glycolysis of hemoglobin in the blood; it is used for long-term glycemic control in diabetic patients and for diagnosis in non-diabetic patients. It is also known that it may indicate the risk of complications in diabetes and reflect the quality of follow-up control of patients. Although HbA1c is below 6% in individuals without diabetes, it may also exceed 10% in individuals who have uncontrolled diabetes [8].

Microangiopathy is regarded as the main mechanism of degenerative diabetes complications observed with retinopathy, nephropathy, and neuropathy [9]. Microangiopathy is also considered as the main factor to cause hearing loss in diabetes [10]. Conducted studies have reported a relationship between HbA1c and hearing loss [11-13]. Our study was aimed to reveal the correlation between HbA1c and hearing loss by comparing the audiometric parameters of the groups.

## Materials and methods

In our study, 724 patients whose HbA1c test parameters were monitored and who were taken to audiological testing between December 2014 and September 2018 were examined. The patients with a history of chronic otitis, ototoxicity, acoustic trauma, malignancy, previous ear surgery, neurological disease, and mental development disorders were excluded from the study. To exclude presbycusis and genetic predisposing factors, patients under the age of 18 and over 65 were not included in the study. We excluded patients older than 65 years in order to avoid confounding effects of age, such as presbycusis. A total of 724 patients in the study population were divided into four groups according to their HbA1c values. One hundred and ninety-two patients with HbA1c values < 4.5 were defined as Group 1, 176 patients between 4.5 and 5 as Group 2, 177 patients between 5 and 6 as Group 3, and 179 patients that were greater than 6 as Group 4. The relationship between the patients’ HbA1c blood result values and pure tone audiological test results were statistically evaluated. HbA1c was measured in venous blood with ethylenediaminetetraacetic acid (EDTA) using the high-performance liquid chromatography (HPLC) method, as well as using the hexokinase method in the Adams™ A1c HA-8180V analyzer (Arkray, Inc., Minneapolis, MN). Pure tone audiometry examinations were performed with the Interacoustics AC40 audiometer (Interacoustics, Eden Prairie, MN) with frequency ranges of 250, 500, 1,000, 2,000, 4,000, and 8,000 Hz. Threshold values were measured separately for the right and left ears. At 250, 500, 1000, 2000, 4000, 8000 Hz, speech recognition threshold (SRT), pure tone averages, and speech discrimination score (SDS) of the individuals were determined for both ears. It was evaluated whether there was a significant difference between the groups for each parameter, including age and gender. The Ankara Yıldırım Beyazıt University, Yenimahalle Government Hospital Ethics Committee approved this study (IRB #11-119).

Statistical analyses

All data were analyzed using the IBM Statistical Package for Social Sciences (SPSS), v. 22 (IBM SPSS Statistics, Armonk, NY). In the analysis of the data, parametric/nonparametric tests were tested first. To decide the normality of the distribution, the kurtosis and skewness values (which are the other assumptions of the Kolmogorov-Smirnov normal distribution and histogram chart) were then used. The Kruskal-Wallis H test was employed to compare two or more unrelated groups. In cases where a difference was found, the pairwise test was used to determine the source of the difference. The pairwise comparison determines significance by making the Bonferroni correction to correct Type 1 alpha error in the Kruskal-Wallis test. To interpret the significance of the obtained values, a significance level of 0.05 was used. In cases where there was a significant difference, 0.008 (0.05/6) was used as a criterion to interpret the significance with the Bonferroni correction to determine where the difference was between the groups.

## Results

Of the 724 patients who participated in the study, 376 were female (51.9%) and 348 (48.1%) were male. The mean age of the patients was found to be 39.82 ± 12.44. There was no significant difference observed between the groups in terms of mean age and gender (Table 1).

**Table 1:** Comparison of Demographic Data Between the Four Groups

Groups	Mean age	Gender (F/M)
Group 1 (HbA1c value < 4.5)	39.82 ± 12.44	87/92
Group 2 (HbA1c value between 4.5 - 5)	39.22 ± 6.02	89/96
Group 3 (HbA1c value between 5 - 6)	38.96 ± 12.56	88/95
Group 4 (HbA1c value greater than 6)	39.01 ± 7.41	84/93
P-value	0.96	-
Total	39.82 ± 12.44	376/348

When Table 2 is examined, a statistically significant difference was identified between the groups in terms of PTT (pure tone threshold) in the right ear according to the HbA1c levels (P ≤ 0.008). In the pairwise comparison test in terms of SDS, PTT, and SRT, the results of the patients in the fourth group were found to be statistically significantly higher than the other three groups.

**Table 2:** Median Values of Audiometric Parameters of the Groups (Right Ears) *p ≤ 0.008 PTT: pure-tone threshold

PTTs	Groups	n	Median Average	Mean Decibel	p-value
Right ear, 250 Hz	1	192	343.52	15.4219	0.16
	2	176	354.07	15.5227	
	3	177	363.09	15.5876	
	4	179	390.56	17.3073	
Right ear, 500 Hz	1	192	336.56	14.6302	< 0.001*
	2	176	345.47	15.1648	
	3	177	360.16	15.0621	
	4	179	409.39	17.0056	
Right ear, 1,000 Hz	1	192	344.53	14.3438	0.09
	2	176	351.55	14.7727	
	3	177	355.2	14.7627	
	4	179	395.8	16.8324	
Right ear, 2,000 Hz	1	192	342.58	15.3646	0.12
	2	176	358.51	15.6534	
	3	177	357.51	15.3390	
	4	179	392.94	18.6704	
Right ear, 4,000 Hz	1	192	339.88	16.2760	< 0.001*
	2	176	353.78	16.8011	
	3	177	354.98	16.3842	
	4	179	402.78	20.2346	
Right ear, 8,000 Hz	1	192	343.38	16.0104	0.08
	2	176	360.22	17.0966	
	3	177	351.94	16.6441	
	4	179	395.69	21.1285	
Right ear	1	192	340.2	15.8333	< 0.001*
	2	176	349.33	16.3864	
	3	177	350.78	15.9096	
	4	179	410.95	19.4525	

When Table 3 is examined, a statistically significant difference was found between the groups in terms of 250 Hz, 500 Hz, 1,000 Hz, 2,000 Hz, 4,000 Hz, and PTT according to HbA1c levels (p ≤ 0.008). As a result of the pairwise comparison test to determine which groups differ in terms of HbA1c levels, it was found that Group 4 was statistically significantly higher in all frequency values and PTT, SDS, SRT compared to Groups 1, 2, and 3 (p ≤ 0.008) (Table 3).

**Table 3:** Median Values of Audiometric Parameters of the Groups (Left Ears) *p ≤ 0.008 PTT: pure-tone threshold

PTT	Groups	n	Median average	Mean Decibel	P-value
Left ear, 250 Hz	1	192	321.54	15.2083	0.001*
	2	176	312.07	14.9886	
	3	177	366.08	15.9492	
	4	179	452.47	17.2067	
Left ear, 500 Hz	1	192	352.58	13.9323	0.001*
	2	176	323.18	13.3466	
	3	177	341.47	13.9831	
	4	179	432.6	15.8101	
Left ear, 1,000 Hz	1	192	355.66	14.1354	0.001*
	2	176	318.19	14.5739	
	3	177	331.36	14.6893	
	4	179	444.36	18.8101	
Left ear, 2,000 Hz	1	192	352.08	15.3177	0.001*
	2	176	314.88	15.8068	
	3	177	344.73	16.2486	
	4	179	438.08	20.5810	
Left ear, 4,000 Hz	1	192	338.04	16.2552	0.001*
	2	176	320.92	17.6477	
	3	177	339.9	17.4802	
	4	179	451,96	24,0056	
Left ear, 8,000 Hz	1	192	338.46	16.6146	0.001*
	2	176	312.89	17.8295	
	3	177	328.34	18.2712	
	4	179	470.84	27.2402	
Left ears	1	192	336.32	15.6615	0.001*
	2	176	323.52	16.7386	
	3	177	337.28	16.0847	
	4	179	453.84	21.1173	

The median values of SRT and SDS also did significantly differ among the four groups both on right and left ears (p < 0.001) (Table 4).

**Table 4:** Median Values of Speech Reception Thresholds (SRTs) and Speech Discrimination Scores (SDSs) of the Groups *p ≤ 0.008 dB: decibel

	Group 1	Group 2	Group 3	Group 4	P-value
Right SRT (dB)	5 (0–15)	5 (0–15)	10 (0–20)	20 (5–40)	< 0.001*
Left SRT (dB)	10 (0–15)	5 (0–15)	10 (0–20)	20 (5–40)	< 0.001*
Right SDS (%)	95 (90–100)	92 (86–100)	88 (76–96)	84 (68–100)	< 0.001*
Left SDS (%)	92 (84-100)	88 (78–100)	84 (70–100)	82 (68–100)	< 0.001*

## Discussion

Hearing losses are defined as a general public health problem since they affect 5% of the general population and cause functional and occupational losses which can lead up to asociality and dementia in individuals [3]. It is stated that age-related hearing losses increase after the fifth decade. However, hearing loss may develop at an earlier age depending on underlying metabolic diseases. Thus, it is important to reveal the causes of hearing loss and to take preventive measures. Diabetes has negative effects on almost completely all systems. Diabetes might also affect vestibular and cochlear systems; unfortunately, the mechanisms of diabetes, the pathophysiology, and characteristics on the cochlear system are not completely resolved. There is a lack of literature about the correlation between diabetes and the features of hearing impairment [11-13]. In addition to studies describing that diabetes-related hearing losses are progressive, bilateral, and involving high frequencies, there are also studies describing loss in all frequencies [14-15].

In our study, we classified patients according to their HbA1c values and compared the results of pure tone audiometry. When the results were analyzed, it was observed that Group 4 had higher losses than the other groups in terms of PTT, SDS, and SRT for the right ear, while the left ear revealed a significantly higher loss in all frequencies, and PTT, SDS, SRT in Group 4 compared to the other groups. It may be incidental to see all the results evident only in the left ear. These results show that there is a relationship between blood HbA1c value and sensorineural hearing loss. The severity of diabetes could be a determining factor for hearing impairment based.on this hypothesis. We performed a comprehensive blood HbA1c parametric level and audiological evaluation and aimed to compare the effects of diabetes on the cochlear system.

There is a theory that diabetes-related hearing impairment will develop mainly at high frequencies since the high-frequency specific areas of the cochlea may be more susceptible to ischemic changes due to microvascular complications [10]. Postmortem studies in patients with uncontrolled diabetes show demyelination in the acoustic nerve and the loss of outer hair cells in the lower basal fold of the cochlea [16]. In addition, when Nemati et al. compared the pure tone audiometry and otoacoustic emission results of 104 patients diagnosed with type 2 diabetes, a higher hearing loss was shown in all frequencies in the pure sound test results of the patient group who were not followed up regularly [13]. In our study, while all frequencies were affected in the left ear, PTT in the right ear was significantly higher in Group 4 than in other groups. The distribution of ages and gender did not differ in all groups. Therefore, the possible confusing factors of age, such as in presbycusis, were eliminated. This can implicate a clinical result in patients with severe to moderate diabetes, according to blood HbA1c parameters, who may, therefore, be at risk of hearing impairment regardless of age.

As a result, we can hypothesize that the diabetes-related hearing losses first affect the basal return of the cochlea. The apical segments of the cochlea are then damaged after the prolonged presence of elevated Hba1c levels so that all frequencies are affected in a long-term process. However, this study is a cross-sectional analysis study, and it should be taken into consideration that a clear mechanism cannot be determined. In this respect, there is a need for prospective studies in which diabetic patients are followed in terms of hearing. In this way, it will be possible to describe the effects of diabetes on hearing more clearly.

Prior studies showed that as the HbA1c value used to monitor the risk of complications in diabetes increases, sensorineural hearing loss also increases [10-12]. In a study conducted by Ooley et al. in 2017, it was revealed that in 175 patients with diabetic retinopathy and whose HbA1c value was known, there was a relationship between the hearing loss and the HbA1c value, as well as the degree of diabetic retinopathy [12]. Similarly, in another study conducted by Nagahama et al. in 2018, the hearing test results of the patient group with an HbA1c value of 7.3 and above revealed significantly higher losses at higher frequencies than other groups (11). In another study, the relationship between HbA1c and hearing loss in non-diabetic patients was evaluated, and it was shown that there was a relationship between HbA1c's high value and hearing loss even in non-diabetic patients [17]. However, Ashkezari et al. stated that there was no correlation between HbA1c and hearing loss in diabetic patients [18]. Nonetheless, in addition to the prior known literature, we expanded the number of patients to achieve a more significant correlation about all frequencies and HbA1c levels. We found that when the HbA1c value was 6 and above, PTT results were evident and increased in both ears. In this study, we found that severe diabetes, according to HbA1c blood levels, might have evident efficacy on all audiometric parameters. According to these results, we especially recommend to endocrinologists that they take a routine audiometric evaluation in those patients with an HbA1c value of 6 and above for hearing loss in follow-up examinations.

Many potential mechanisms can be considered for hearing loss in diabetes patients regarding the severity of diabetes. In the study by Kim et al., fasting plasma glucose (FPG) and HbA1c were found to be independent risk factors for microvascular complications [19]. Considering that other diabetes complications, such as retinopathy, nephropathy, and neuropathy caused due to microangiopathy, can be reduced and prevented by controlling hyperglycemia, there is a possibility to prevent these hearing losses with routine controls.

Our study had certain limitations. Since our study was a cross-sectional study that did not contain information about the duration of the disease, and other systemic causes and blood parameters were not included to define the relationship between the level of HbA1c in the blood and the development of hearing loss, prospective studies involving a larger number of patients and more variables are needed.

## Conclusions

This study is a source for studies investigating the possible relationship of diabetes with sensorineural hearing loss and cochlear complications by pure sound audiometry and HbA1c value, age, and gender. We found that the pure audio audiological test results showed involvement not only in high frequency but also in low frequencies when the HbA1c values increase in patients. Consequently, if optimal conditions are provided, patients with high HbA1c values should be subjected to tighter control in terms of audiological tests. It is recommended not to overlook that hearing loss of patients with high HbA1c levels may be progressive. In these patients, routine annual audiometry and treatment for hyperglycemia are suggested in order to prevent the progression of hearing loss. We would recommend future prospective studies be carried out in order to study diabetic patients in terms of hearing in order to describe the effects of diabetes on hearing more clearly.
